# Bacterial Microcompartments Coupled with Extracellular Electron Transfer Drive the Anaerobic Utilization of Ethanolamine in Listeria monocytogenes

**DOI:** 10.1128/mSystems.01349-20

**Published:** 2021-04-13

**Authors:** Zhe Zeng, Sjef Boeren, Varaang Bhandula, Samuel H. Light, Eddy J. Smid, Richard A. Notebaart, Tjakko Abee

**Affiliations:** a Food Microbiology, Wageningen University and Research, Wageningen, the Netherlands; b Laboratory of Biochemistry, Wageningen University and Research, Wageningen, the Netherlands; c Department of Molecular and Cell Biology, University of California, Berkeley, Berkeley, California, USA; d Department of Microbiology, The University of Chicago, Chicago, Illinois, USA; California State University, Fresno

**Keywords:** *Listeria monocytogenes*, anaerobic catabolic pathways, electron transport, microcompartment

## Abstract

Ethanolamine (EA) is a valuable microbial carbon and nitrogen source derived from cell membranes. EA catabolism is suggested to occur in a cellular metabolic subsystem called a bacterial microcompartment (BMC), and the activation of EA utilization (*eut*) genes is linked to bacterial pathogenesis. Despite reports showing that the activation of *eut* is regulated by a vitamin B_12_-binding riboswitch and that upregulation of *eut* genes occurs in mice, it remains unknown whether EA catabolism is BMC dependent in Listeria monocytogenes. Here, we provide evidence for BMC-dependent anaerobic EA utilization via metabolic analysis, proteomics, and electron microscopy. First, we show vitamin B_12_-induced activation of the *eut* operon in L. monocytogenes coupled to the utilization of EA, thereby enabling growth. Next, we demonstrate BMC formation connected with EA catabolism with the production of acetate and ethanol in a molar ratio of 2:1. Flux via the ATP-generating acetate branch causes an apparent redox imbalance due to the reduced regeneration of NAD^+^ in the ethanol branch resulting in a surplus of NADH. We hypothesize that the redox imbalance is compensated by linking *eut* BMCs to anaerobic flavin-based extracellular electron transfer (EET). Using L. monocytogenes wild-type, BMC mutant, and EET mutant strains, we demonstrate an interaction between BMCs and EET and provide evidence for a role of Fe^3+^ as an electron acceptor. Taken together, our results suggest an important role of BMC-dependent EA catabolism in L. monocytogenes growth in anaerobic environments like the human gastrointestinal tract, with a crucial role for the flavin-based EET system in redox balancing.

**IMPORTANCE** Listeria monocytogenes is a foodborne pathogen causing severe illness, and as such, it is crucial to understand the molecular mechanisms contributing to pathogenicity. One carbon source that allows L. monocytogenes to grow in humans is ethanolamine (EA), which is derived from phospholipids present in eukaryotic cell membranes. It is hypothesized that EA utilization occurs in bacterial microcompartments (BMCs), self-assembling subcellular proteinaceous structures and analogs of eukaryotic organelles. Here, we demonstrate that BMC-driven utilization of EA in L. monocytogenes results in increased energy production essential for anaerobic growth. However, exploiting BMCs and the encapsulated metabolic pathways also requires the balancing of oxidative and reductive pathways. We now provide evidence that L. monocytogenes copes with this by linking BMC activity to flavin-based extracellular electron transfer (EET) using iron as an electron acceptor. Our results shed new light on an important molecular mechanism that enables L. monocytogenes to grow using host-derived phospholipid degradation products.

## INTRODUCTION

Pathogens have evolved mechanisms to utilize specific metabolites as carbon sources to sidestep nutritional competition with commensal bacteria in the human gastrointestinal (GI) tract (1–4). Ethanolamine (EA), a product of the breakdown of phosphatidylethanolamine from eukaryotic cell membranes, is such a metabolite and is abundant in the human GI tract (5, 6). It has been shown that some species in the GI tract, like Salmonella enterica, Enterococcus faecalis, and Clostridium perfringens, can use EA as a carbon source, while for some other human pathogens, including Listeria monocytogenes, the putative use of EA as a substrate was postulated based on the presence of a similar gene cluster (5–7). The ability to utilize EA is encoded by the ethanolamine utilization (*eut*) operon (6–8). EA is converted to acetaldehyde and ammonia by the ethanolamine ammonia lyase EutBC (6, 9, 10). Acetaldehyde can be catabolized to ethanol by the alcohol dehydrogenase EutG (8) or to acetyl-CoA by the acetaldehyde dehydrogenase EutE (6, 11). Acetyl-CoA can be degraded to acetate with ATP production by the phosphotransacetylase EutD (12) and an alternative acetate kinase, EutQ (13). Alternatively, acetyl-CoA can be catabolized in the tricarboxylic acid cycle or the glyoxylate cycle or used for lipid biosynthesis (6). According to current models, EA is catabolized to acetate and ethanol in a molar ratio of 1:1 via the oxidative ATP-producing branch and reductive NAD^+^-regenerating branch (6). Interestingly, previous studies showed that EA confers a marked anaerobic growth advantage on Salmonella enterica serovar Typhimurium only in the presence of tetrathionate, acting as an alternative electron acceptor via tetrathionate reductase (6, 14–16), and the mutant lacking the tetrathionate reductase showed a decreasing colonization capacity in a mouse colitis model, which points to a role for anaerobic electron transfer in EA catabolism contributing to the growth of S. enterica in the lumen of the inflamed intestine (15). Anaerobic EA catabolism in L. monocytogenes, including possible roles for anaerobic respiration, has not been studied. Notably, L. monocytogenes lacks tetrathionate reductase, but anaerobic electron transfer with fumarate reduction via membrane-bound fumarate reductase (17) and the recently described flavin-based extracellular electron transfer (EET) with Fe^3+^ as an electron acceptor (18) could act as substitutes in bacterial microcompartment (BMC)-dependent EA catabolism.

The enzymes of the indicated EA pathway are present in a BMC, and structural shell proteins that constitute the BMC building blocks are encoded by genes in the *eut* cluster (19). BMCs consist of a capsule of semipermeable shell proteins and encapsulated enzymes of metabolic pathways that liberate toxic intermediates in the lumen of the capsules (19–21). In the formation of BMCs, so-called encapsulation peptides, 10- to 20-residue-long hydrophobic α-helices in the N termini of some core enzymes, play a key role in the encapsulation mechanism (22, 23). In our previous study, evidence was provided for a role of BMC-dependent utilization of 1,2-propanediol in L. monocytogenes supporting anaerobic growth and metabolism, and the encapsulated enzymes PduD, PduL, and PduP were found to contain encapsulation peptides (24). Expression of the *eut* operon in L. monocytogenes is under the regulation of the two-component regulators EutVW sequestrated by a vitamin B_12_-binding riboswitch (25, 26). Upregulation of the *eut* operon has been found in L. monocytogenes grown on vacuum-packed cold-smoked salmon and in cocultures with cheese rind bacteria, which suggests a possible role of the *eut* operon in the adaptation of L. monocytogenes to available nutrient sources (27, 28). The L. monocytogenes *eut* operon exhibited increased expression inside the host cell, and the loss of one of the key enzymes, ethanolamine ammonia lyase (EutB), caused a defect in intracellular growth (29).

Here, we show by using metabolic analysis, transmission electron microscopy (TEM), and proteomics that L. monocytogenes forms *eut* BMCs and utilizes EA as a carbon source under anaerobic conditions with the end products acetate and ethanol in a molar ratio of 2:1. We demonstrate that the resulting redox imbalance is compensated by the flavin-based EET system by comparative growth and metabolic analyses of L. monocytogenes wild-type, BMC mutant, and EET mutant strains. Our results suggest an important role of anaerobic BMC-dependent EA catabolism in the physiology of L. monocytogenes, with a crucial role for the flavin-based EET system in redox balancing.

## RESULTS

### EA utilization facilitates anaerobic growth.

**FIG 1:**
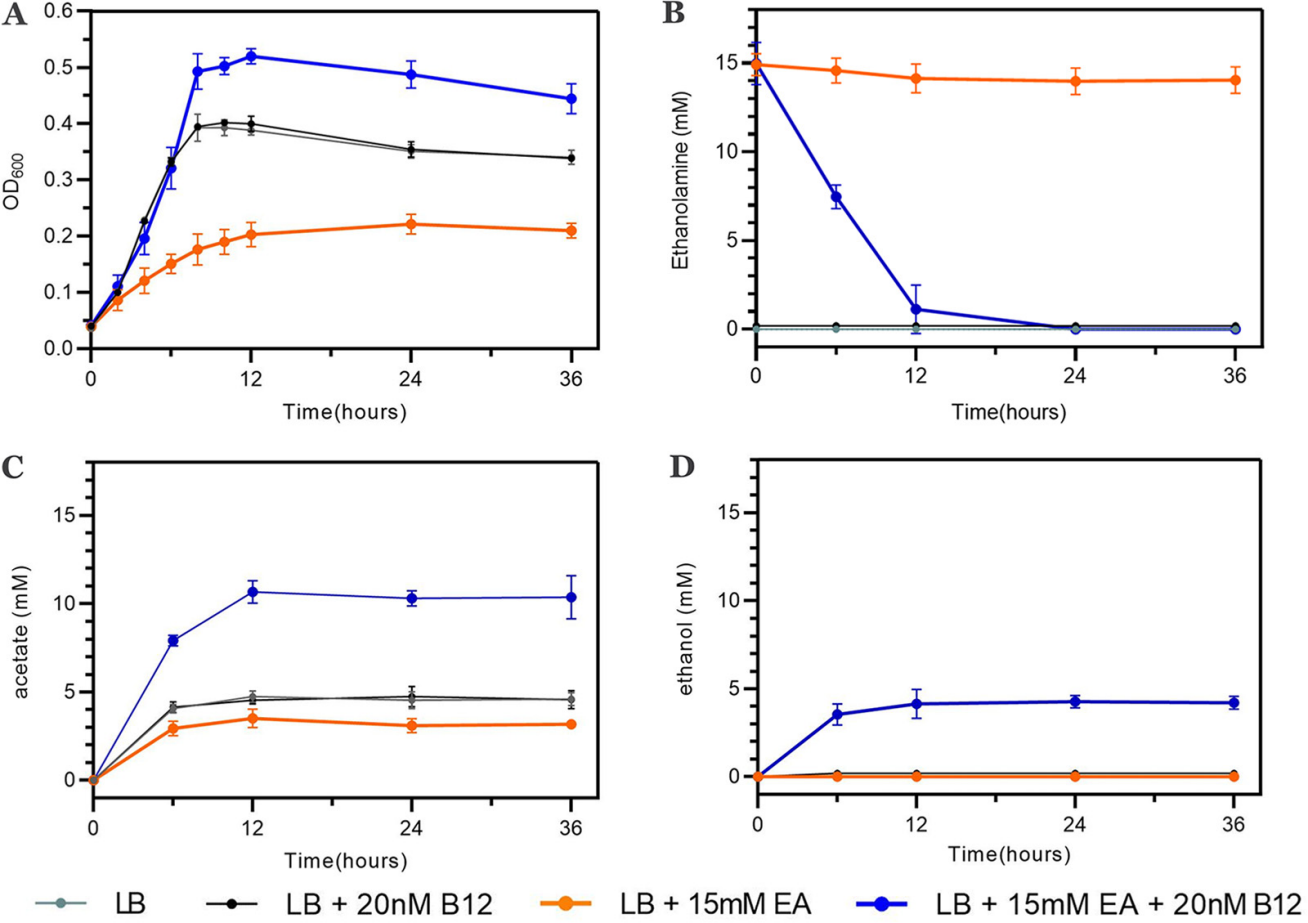
Anaerobic growth and EA catabolism of L. monocytogenes EGDe in LB medium. (A) Impact of EA and/or vitamin B_12_ on anaerobic growth of L. monocytogenes EGDe; (B) EA utilization; (C) acetate production; (D) ethanol production. Lines represent different growth conditions. Results from three independent experiments are expressed and visualized as means and standard errors.

To find out whether EA utilization stimulates anaerobic growth, we added EA (15 mM) to Luria broth (LB) medium in line with previous work in *Salmonella* species (11, 30). The culture medium also contained 20 nM vitamin B_12_ for the activation of the *eut* operon in L. monocytogenes (25). L. monocytogenes EGDe cultures grown under *eut*-induced conditions (LB with EA and B_12_) reached significantly higher optical density at 600 nm (OD_600_) values after 8 h of incubation than cultures under control conditions (LB and LB with B_12_) and those under *eut*-noninduced conditions (LB with EA) (Fig. 1A). EA under *eut*-induced conditions was fully utilized within the first 24 h, while no significant EA utilization was observed under *eut*-noninduced conditions (Fig. 1B). Notably, under *eut*-induced conditions, EA was converted into acetate and ethanol (Fig. 1C and D), while under *eut*-noninduced conditions, only acetate was produced, conceivably originating from the metabolism of other compounds in LB. From this comparative analysis of acetate production, we derive a molar ratio of acetate to ethanol of approximately 2:1. Taken together, the utilization of EA with the production of acetate and ethanol contributes to the anaerobic growth of L. monocytogenes EGDe under *eut*-induced conditions.

### Ratio of ethanol to acetate production.

**FIG 2:**
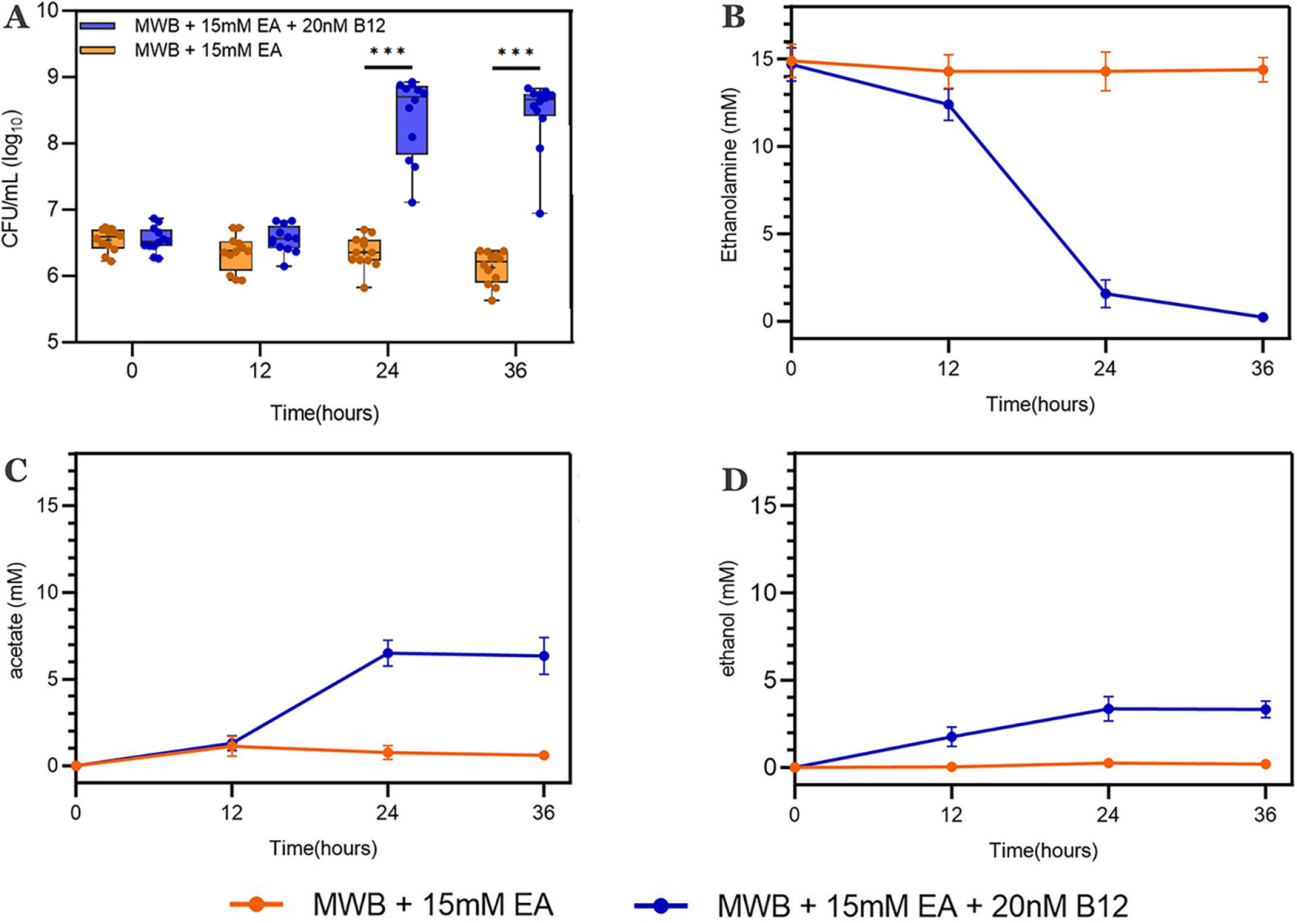
Anaerobic growth and EA catabolism of L. monocytogenes EGDe in MWB defined medium with EA as the sole carbon source. (A) Impact of EA and/or vitamin B_12_ on CFU of L. monocytogenes EGDe. Results from three independent experiments with four technical repeats are expressed as means and standard errors. Statistical significance is indicated (***, *P* < 0.001 [by a Holm-Sidak *t* test]). (B) EA utilization. (C) Acetate production. (D) Ethanol production. Cells were grown in MWB plus 15 mM EA without B_12_ and with 20 nM B_12_. Error bars in panels B to D indicate results from three independent experiments expressed as means and standard errors.

To clarify whether L. monocytogenes EGDe can utilize EA as a sole carbon source, we examined EA utilization and its impact on anaerobic growth in the defined medium Modified Welshimer’s broth (MWB) in the absence of any other carbon sources (31). L. monocytogenes EGDe inoculated under *eut*-induced conditions (MWB with EA and vitamin B_12_) showed an ∼100-fold increase in cell counts after 24 h, while no significant increase in cell counts was observed under noninduced conditions (MWB with EA) (Fig. 2A). Microscopy analysis of samples showed the absence of chains of cells, excluding the option that the increase in CFU is due to the disintegration of chains of cells. During the anaerobic growth of L. monocytogenes EGDe, 15 mM EA was converted into about 6.6 mM acetate and 3.4 mM ethanol under *eut*-induced conditions, while no significant degradation of EA was observed under noninduced conditions (Fig. 2B to D). Through calculation of the carbon mass balance, part of EA (approximately 5 mM) is conceivably further catabolized in the γ-aminobutyrate (GABA) shunt (32) or used for lipid biosynthesis via the intermediate acetyl-CoA (6). The utilization of EA under *eut*-induced conditions in the defined medium MWB provides evidence that EA can act as a sole carbon source supporting the anaerobic growth of L. monocytogenes EGDe. Notably, the observed molar ratio of acetate to ethanol of 2:1 suggests an apparent redox imbalance due to higher flux via the NADH- and ATP-producing acetate branch and reduced regeneration of NAD^+^ in the ethanol branch resulting in a surplus of NADH.

### Upregulated expression of the *eut* operon, including enzymes and structural shell proteins.

**FIG 3:**
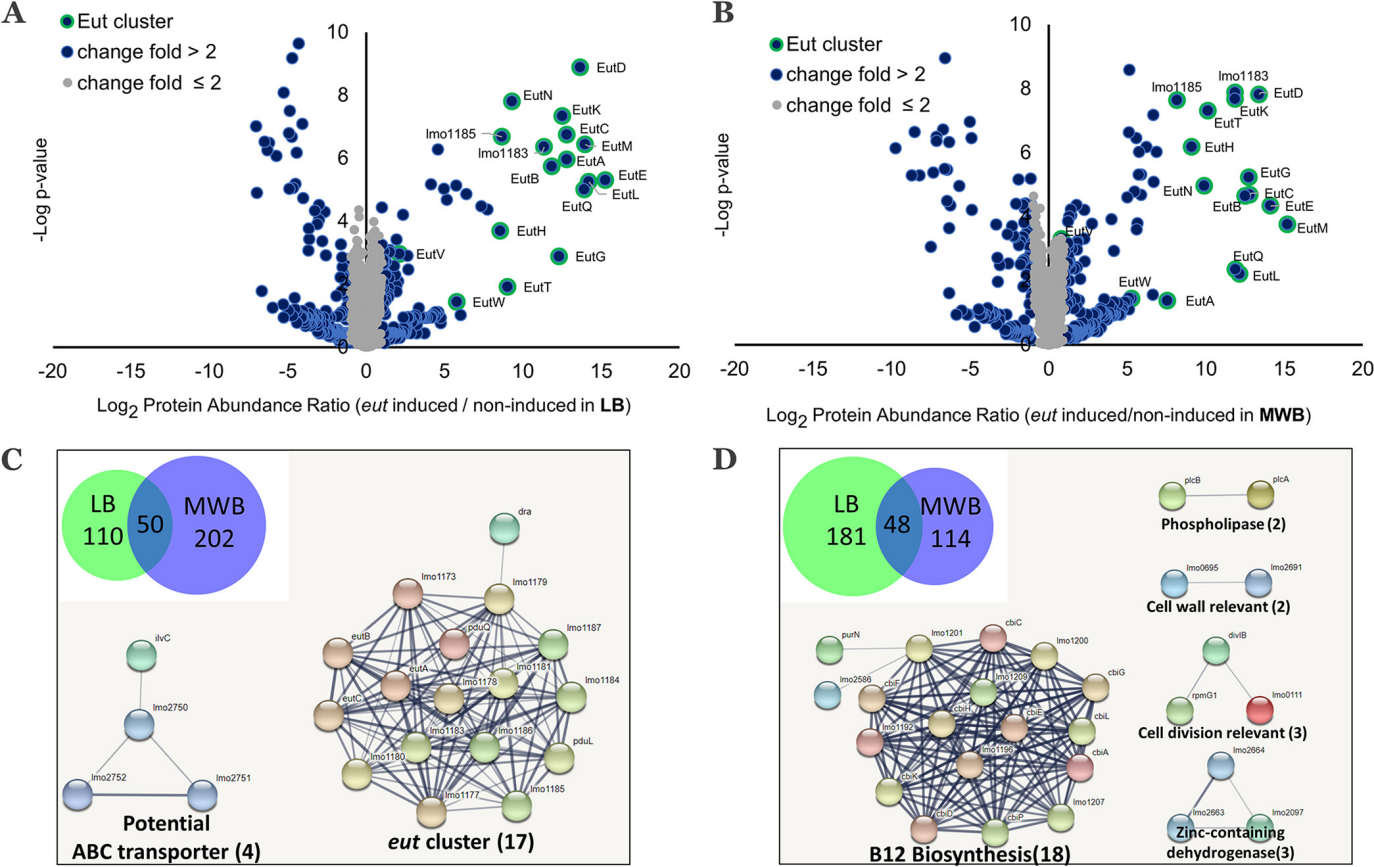
Proteomics analysis of *eut*-induced and noninduced L. monocytogenes EGDe in LB medium and MWB defined medium. (A and B) Proteomic volcano plot of *eut*-induced cells (EA and vitamin B_12_ added) compared to noninduced cells (EA added only) in LB medium (A) and MWB medium (B). (C and D) Venn diagram of 50 overlapping upregulated proteins (C) 48 overlapping downregulated proteins (D) for LB medium (green) and MWB medium (blue) and corresponding STRING protein-protein interactions. Nodes represent proteins, and lines represent interactions.

In order to study the expression of the *eut* operon and the way in which it is embedded in cell physiology, we performed proteomics to compare L. monocytogenes cells grown in LB and MWB medium under *eut*-induced conditions and noninduced conditions. Analyses of the complete list of identified proteins, the proteins’ expression levels, and subsequent *t* test results and *P* values are shown in Table S2 in the supplemental material for LB-grown and in Table S3 for MWB-grown cells. For LB, we identified 1,891 total proteins where 161 proteins are upregulated more than 2-fold and 229 proteins are downregulated more than 2-fold under *eut*-induced conditions compared to noninduced conditions (Fig. 3A). Among these 161 upregulated proteins, the top 15 proteins are all encoded in the *eut* operon, i.e., EutGABCLKEMTDNHQ, lmo1183, and lmo1185, while the other two proteins, EutVW (33), involved in *eut* operon regulation are also included. For MWB, 1,736 proteins were identified, of which 253 proteins are upregulated more than 2-fold and 162 proteins are downregulated more than 2-fold under *eut*-induced conditions compared to noninduced conditions (Fig. 3B). In line with the LB data, the top 15 proteins are all encoded in the *eut* operon. Analysis of the upregulated proteins in LB and MWB media shows 50 proteins that overlap between the conditions, pointing to a prominent role under *eut*-induced conditions compared to noninduced conditions. Among these 50 proteins, 17 proteins are linked to *eut*, of which 16 proteins are encoded in the *eut* operon, and one gene, *dra* (deoxyribose-phosphate aldolase), is predicted to have an interaction with the acetaldehyde dehydrogenase *eutE* (*lmo1179*) (according to STRING analysis) (Fig. 3C; Table S4). Another group of overlapping genes comprises potential ABC transporters, including *lmo2751-lmo2752* (ABC transporter ATP-binding protein) (34), and *ilvC* (*lmo1986*) (NADP^+^-based ketol acid reductoisomerase) (35). The analysis of downregulated proteins in LB and MWB showed an overlap of 48 proteins, with 18 proteins linked to vitamin B_12_ biosynthesis, which indicates that adding vitamin B_12_ to the medium represses the genes involved in vitamin B_12_ biosynthesis (Fig. 3D; Table S5). The overlap in downregulated proteins also showed enrichment in phospholipases (*plcA* and *plcB*), putative cell wall-relevant proteins (*lmo2691* [putative peptidoglycan hydrolase autolysin] and *lmo0695* [similar to the flagellar hook length control protein FliK]), cell division-relevant proteins (*dilvB*, *rpmG1*, and *lmo0111*) (Table S5), and zinc-containing dehydrogenases (*lmo2663*, *lmo2664*, and *lmo2097*). To summarize, the significant upregulation of the *eut* operon at the proteomic level, including structural shell proteins, strongly supports that BMC-dependent EA utilization is processed by enzymes and structural shell proteins of the *eut* operon.

10.1128/mSystems.01349-20.4 TABLE S2 Protein profiling of *eut*-induced compared with noninduced L. monocytogenes EGDe in LB medium. Shown are the UniProt protein identifiers, *x* values representing log_2_ protein abundance ratios (*eut* induced/noninduced in LB), *y* values representing −log_10_ *P* values (*eut* induced/noninduced in LB), NCBI protein annotations, and NCBI gene identifiers. Download Table S2, XLSX file, 0.1 MB. Copyright © 2021 Zeng et al. 2021 Zeng et al. https://creativecommons.org/licenses/by/4.0/ This content is distributed under the terms of the Creative Commons Attribution 4.0 International license.

10.1128/mSystems.01349-20.5 TABLE S3 Protein profiling of *eut*-induced compared with noninduced L. monocytogenes EGDe in MWB medium. Shown are the UniProt protein identifiers, *x* values representing log_2_ protein abundance ratios (*eut* induced/noninduced in MWB), *y* values representing −log_10_ *P* values (*eut* induced/noninduced in MWB), NCBI protein annotations, and NCBI gene identifiers. Download Table S3, XLSX file, 0.1 MB. Copyright © 2021 Zeng et al. 2021 Zeng et al. https://creativecommons.org/licenses/by/4.0/ This content is distributed under the terms of the Creative Commons Attribution 4.0 International license.

10.1128/mSystems.01349-20.6 TABLE S4 Overlapping proteins of LB upregulated proteins and MWB upregulated proteins (>2-fold). Shown are the UniProt protein identifiers, KEGG gene identifiers, NCBI protein annotations, and STRING interactions. Download Table S4, XLSX file, 0.01 MB. Copyright © 2021 Zeng et al. 2021 Zeng et al. https://creativecommons.org/licenses/by/4.0/ This content is distributed under the terms of the Creative Commons Attribution 4.0 International license.

10.1128/mSystems.01349-20.7 TABLE S5 Overlapping proteins of LB downregulated proteins and MWB downregulated proteins (>2-fold). Listed are the UniProt protein identifiers, KEGG gene identifiers, NCBI protein annotations, and STRING interactions. Download Table S5, XLSX file, 0.01 MB. Copyright © 2021 Zeng et al. 2021 Zeng et al. https://creativecommons.org/licenses/by/4.0/ This content is distributed under the terms of the Creative Commons Attribution 4.0 International license.

### BMC structures support BMC-dependent EA catabolism.

**FIG 4:**
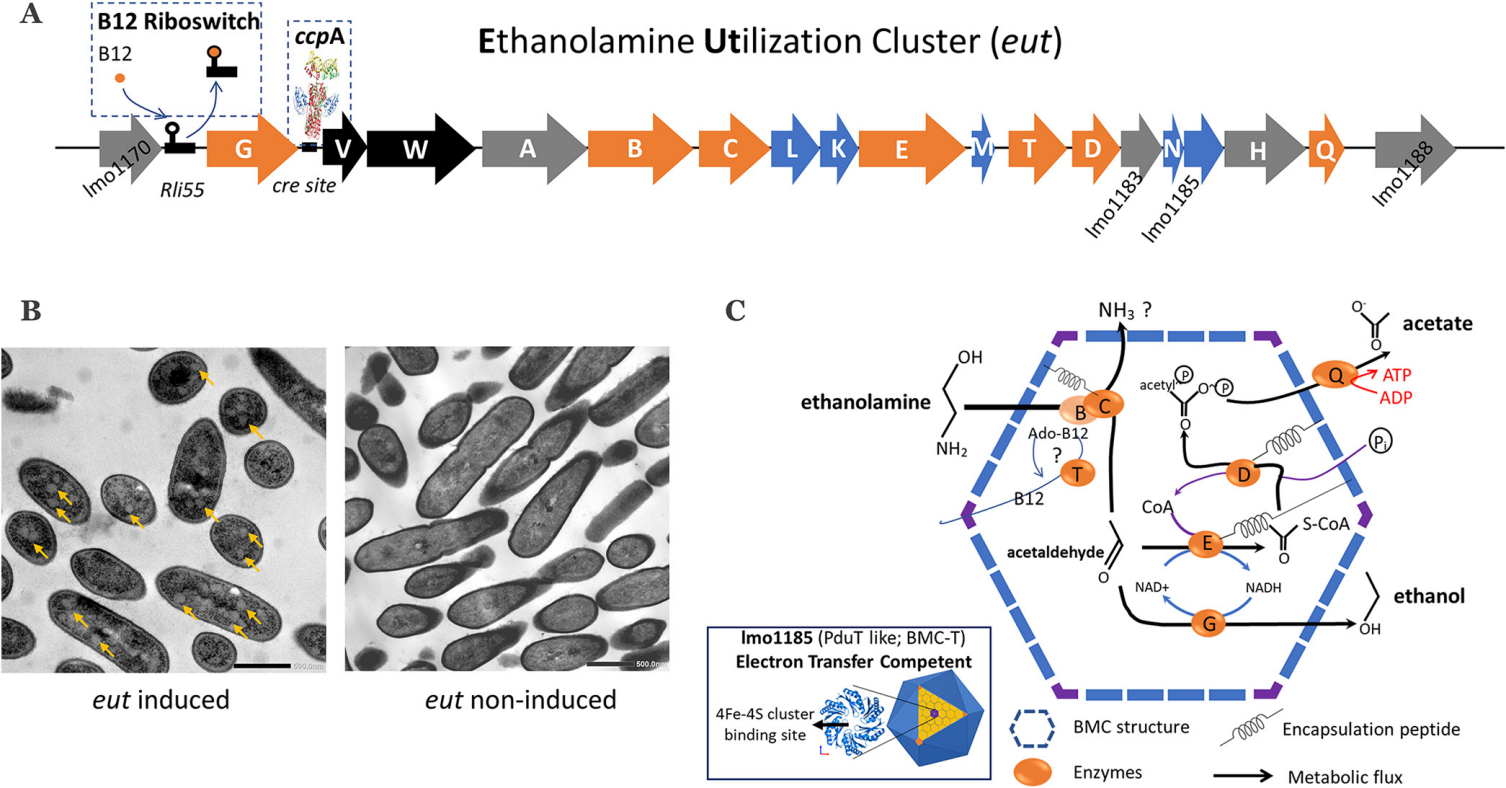
Overview of the BMC-dependent EA catabolism model of L. monocytogenes. (A) Analysis of the *eut* operon (for details, see Table S1 in the supplemental material). Characters in orange represent Eut enzymes, those in blue represent BMC shell proteins, those in black represent the two-component regulation system, and those in gray represent unannotated proteins. The vitamin B_12_ riboswitch and CcpA *cre*-binding site are indicated. (B) TEM visualization of BMCs in *eut*-induced (left) (yellow arrows point to BMCs) and noninduced (right) cells. (C) Model of BMC-dependent EA catabolism. EutBC, ethanolamine ammonia lyase; EutD, phosphotransacetylase; EutE, acetaldehyde dehydrogenase; EutG, alcohol dehydrogenase; EutQ, acetate kinase; EutT, corrinoid cobalamin adenosyltransferase. Putative encapsulation peptides are indicated. The zoomed-in part shows the prediction of the potential [4Fe-4S] cluster-binding site in Eut shell protein lmo1185, which is highly similar to that previously reported for the PduT shell protein by Pang et al. (39). See the text for details.

To further confirm the presence of BMCs in *eut*-induced cells, we used transmission electron microscopy (TEM) and compared thin sections of both *eut*-induced and noninduced L. monocytogenes EGDe cells. The *eut*-induced cells clearly contain BMC-like structures with a diameter of approximately 50 to 80 nm, which are not present in noninduced cells (Fig. 4B). Notably, the identified structures strongly resemble those in TEM pictures of BMCs in S. enterica and Escherichia coli (36, 37) and that of recently reported BMCs found in *pdu*-induced L. monocytogenes (24). Taking the metabolic, proteomic, and TEM data together, we conclude that the cytosolic BMC-like structures in L. monocytogenes EGDe are involved in EA utilization under anaerobic conditions. Here, based on the knowledge of the *eut* operon, we propose a model of BMC-dependent EA catabolism. The *eut* operon in L. monocytogenes EGDe contains 17 genes and is most likely under the regulation of the two-component regulators EutVW sequestrated by the binding of vitamin B_12_ to the riboswitch rli55 (25). In front of EutVW, we found a previously predicted *cre* site for the binding of the carbon control protein CcpA, pointing to catabolite repression control of the L. monocytogenes *eut* cluster (38). Five *eut* genes, *eutLKMN* and *lmo1185*, are predicted to be structural shell proteins of the BMC (Fig. 4A). EutK and EutM are the hexameric shell protein (BMC-H) consisting of one Pfam00936 domain, while EutL and lmo1185 are the trimeric shell proteins (BMC-T) with two fused Pfam00936 domains (Fig. S1). Notably, the trimeric assembly of lmo1185 forms a flat approximately hexagonally shaped disc with a central pore that is suitable for a [4Fe-4S] cluster (39). Furthermore, EutN is the pentameric shell protein (BMC-P) consisting of one Pfam03319 domain (Fig. S1). The BMC is assembled by these three types of shell proteins, BMC-H, BMC-T, and BMC-P (19, 20). Based on the previous BMC-dependent EA catabolism model in S. enterica (5), we propose a model for L. monocytogenes with putative encapsulation peptides supporting the recruitment of selected Eut enzymes to the BMC (Fig. 4C). We predict a specific hydrophobic α-helix in the N terminus of the encapsulated enzymes EutC, EutD, and EutE (Fig. S2), similar to those of the previously described BMC-encapsulated proteins in S. enterica and *pdu* BMCs in L. monocytogenes (22, 24). EA is split into acetaldehyde and ammonia by the ethanolamine ammonia lyase EutBC (6, 9, 10). Acetaldehyde can be converted into ethanol by the alcohol dehydrogenase EutG (8) or into acetyl-CoA by the acetaldehyde dehydrogenase EutE (6, 11). Acetyl-CoA can be converted into acetyl-phosphate and subsequently acetate and ATP by the phosphotransacetylase EutD (12) and an alternative acetate kinase, EutQ, respectively (13).

10.1128/mSystems.01349-20.1 FIG S1 Homologous protein structures and domain architectures of shell proteins in the *eut* operon. (Left) Homologous protein structures of EutL, PduT, EutM, and EutN in other bacteria from the Protein Data Bank in Europe (PDBe). PDBe identifiers for each protein are listed at the top of the protein structures. (Right) Domain architectures of shell proteins in the *eut* operon of L. monocytogenes EGDe analyzed using the UniProtKB database. Download FIG S1, DOCX file, 0.4 MB. Copyright © 2021 Zeng et al. 2021 Zeng et al. https://creativecommons.org/licenses/by/4.0/ This content is distributed under the terms of the Creative Commons Attribution 4.0 International license.

10.1128/mSystems.01349-20.2 FIG S2 Prediction of N-terminal encapsulation peptides. The predicted N-terminal encapsulation peptides of EutC, EutD, and EutE were analyzed by Jpred4, with alpha helices marked as red tubes and sheets marked as green arrows. Jnetconf, confidence estimation for the prediction, with high scores indicating high confidence; Jnetsol25, solvent accessibility, where B means buried and “−” means nonburied at a 25% cutoff. Download FIG S2, DOCX file, 0.5 MB. Copyright © 2021 Zeng et al. 2021 Zeng et al. https://creativecommons.org/licenses/by/4.0/ This content is distributed under the terms of the Creative Commons Attribution 4.0 International license.

10.1128/mSystems.01349-20.3 TABLE S1 Analysis of the *eut* operon. Shown are the NCBI gene identifiers, UniProt protein identifiers, gene name, protein family membership with InterPro identifiers, prediction of shell proteins by HMMER, and prediction of encapsulation peptides by Jpred4. Download Table S1, XLSX file, 0.01 MB. Copyright © 2021 Zeng et al. 2021 Zeng et al. https://creativecommons.org/licenses/by/4.0/ This content is distributed under the terms of the Creative Commons Attribution 4.0 International license.

### Flavin-based EET linked with BMCs maintains redox balance of EA catabolism.

**FIG 5:**
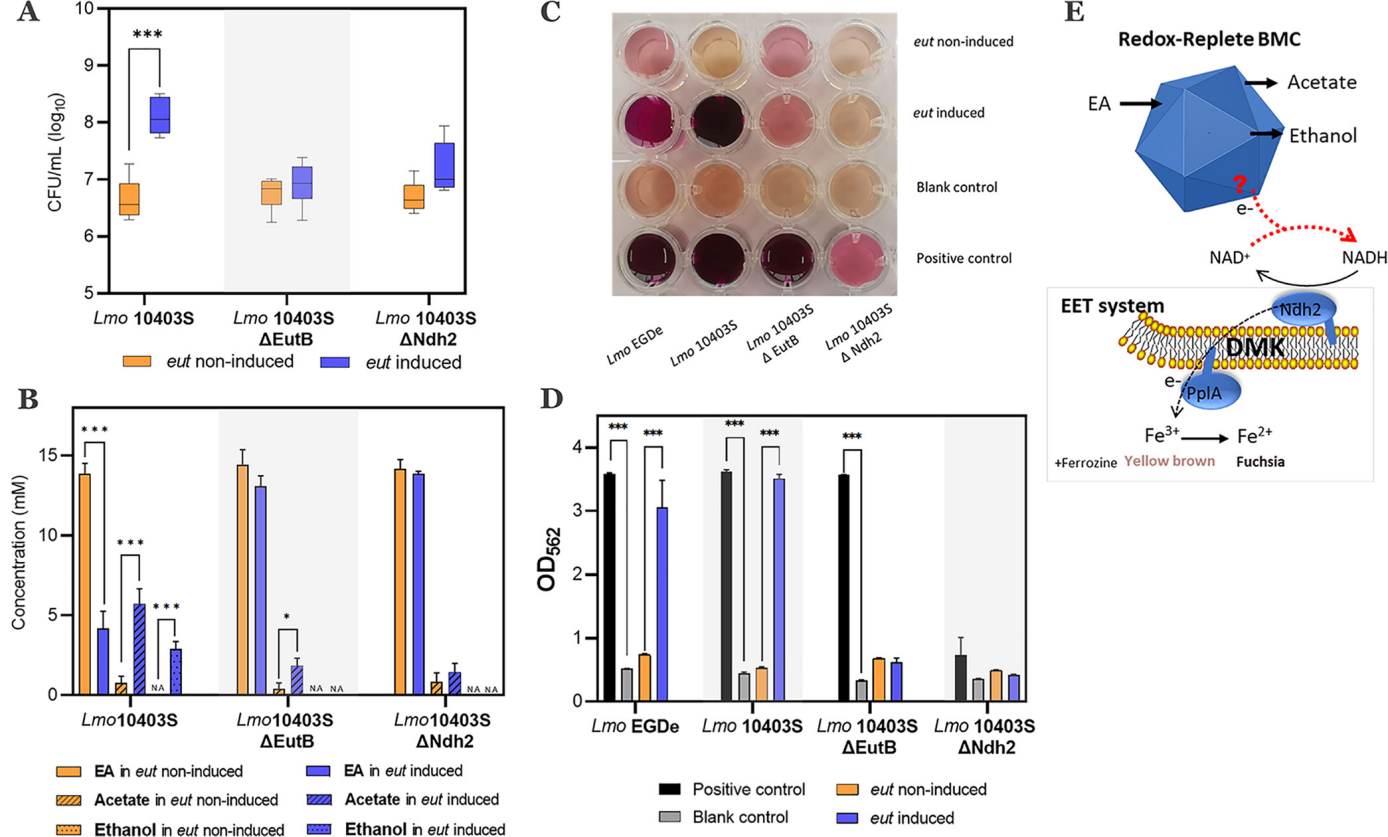
BMC-dependent EA catabolism is coupled to flavin-based EET. (A) Impact of EA and/or vitamin B_12_ on CFU. (B) EA catabolism. Experiments in panels A and B were performed with L. monocytogenes 10403S and mutant strains grown anaerobically for 24 h with an initial inoculum of 6.5 ± 0.1 log_10_ CFU/ml in MWB with 15 mM EA and 20 nM B_12_ (*eut* induced) or 15 mM EA (noninduced). (C) Colorimetric change of the ferric reductase assay. (D) OD_562_ measurements of the ferric reductase assay. Experiments in panels C and D were performed with L. monocytogenes EGDe, 10403S, and mutant strains grown anaerobically for 24 h in MWB with 15 mM EA and 20 nM B_12_ (*eut* induced), 15 mM EA (noninduced), 15 mM glucose (positive control), or no added substrate (blank control). Results in panels A, B, and D are from three independent experiments and are expressed as means and standard errors. Statistical significance is indicated (***, *P* < 0.001; *, *P* < 0.05 [by a Holm-Sidak *t* test]). (E) Proposed model of electron transfer from the BMC to the EET system. The blue geometric block represents the BMC. CM represents the cytoplasmic membrane. Ndh2, PplA, and DMK (demethylmenaquinone) represent EET with Fe^3+^ as an electron acceptor (see the text for details).

The observed unbalanced production of acetate and ethanol in a 2:1 molar ratio suggests a surplus of NADH, and this requires additional NAD^+^ regeneration reactions to restore the redox balance. As discussed above, the BMC is a redox-replete compartment that can generate reductants internally or facilitate the transfer of electrons from the cytosol across the shell (6, 40). Recently, it has been shown that L. monocytogenes uses a distinctive anaerobic flavin-based EET mechanism to deliver electrons to iron (Fe^3+^) or to fumarate via membrane-bound fumarate reductase (18). In our study, MWB defined medium indeed contains ferric citrate and flavin, and proteomic analysis of L. monocytogenes EGDe grown in this medium identified *lmo2637* encoding an EET-linked lipoprotein, PplA, and *lmo2638*, encoding an EET-linked NADH dehydrogenase, Ndh2 (18) (Table S3). Next, we tested the hypothesis that anaerobic EET could play a role in BMC-dependent EA utilization using L. monocytogenes 10403S wild-type and mutant strains. Notably, *eut*-induced L. monocytogenes 10403S showed a growth benefit from utilizing EA similar to that of *eut*-induced L. monocytogenes EGDe (Fig. 5A and B). Moreover, the mutant strains L. monocytogenes 10403S Δ*eutB*, lacking ethanolamine ammonia lyase, and Δ*ndh2*, lacking EET-linked NADH dehydrogenase, are both impaired for EA utilization (Fig. 5B), suggesting an involvement of the EET system in EA utilization and thereby a link with BMCs. Next, we used a ferrozine-based colorimetric assay to determine electron transfer between the BMC and the EET system following L. monocytogenes EA utilization in MWB medium. In this assay, electrons generated from EA catabolism are transferred to Fe^3+^, generating Fe^2+^, where the binding of ferrozine to the differently charged Fe molecules results in a colorimetric change from yellow-brown to fuchsia, respectively. The colorimetric changes were shown for L. monocytogenes EGDe and L. monocytogenes 10403S under *eut*-induced conditions following EA utilization, while *eut* mutant strain L. monocytogenes 10403S Δ*eutB* and EET mutant strain L. monocytogenes 10403S Δ*ndh2* showed no colorimetric changes (Fig. 5C). OD_562_ measurements indicated significantly higher ferric iron reductase activity under *eut*-induced conditions than under noninduced conditions for L. monocytogenes EGDe and L. monocytogenes 10403S (Fig. 5D). Taken together, these results provide evidence for a link between *eut* BMCs and the EET system via the regeneration of NAD^+^ by NADH oxidation in EET using Fe^3+^ as an electron acceptor (Fig. 5E). The action of EET as an alternative reductive pathway next to the ethanol branch thus stimulates anaerobic growth by enhanced flux via the ATP-generating acetate branch in BMC-facilitated EA catabolism.

## DISCUSSION

This study provides evidence for the activation of BMC-dependent ethanolamine (EA) utilization in L. monocytogenes under anaerobic conditions in LB and MWB medium containing EA and vitamin B_12_. By using metabolic analysis, proteomics, and electron microscopy, we demonstrated the formation of BMCs in conjunction with EA catabolism with the production of acetate and ethanol in a 2:1 molar ratio. Selected genes in the *eut* operon encode structural shell proteins that form the respective *eut* BMCs (19). Previous studies showed that BMCs for EA catabolism in S. enterica and E. faecalis are composed of five structural shell proteins, EutS, EutM, EutK, EutL, and EutN (6, 19, 21). Notably, the L. monocytogenes *eut* operon also encodes five putative shell proteins, EutM, EutK, EutL, EutN, and lmo1185, and combined with the visualization of BMC structures by TEM and our proteomic data, we conclude that *eut* BMCs are composed of these five indicated structural shell proteins. Apparently, EutS is not essential for BMC assembly in L. monocytogenes. EutS is hexameric BMC shell protein with a Pfam00936 domain, and it is conceivable that the function of EutS is taken over by EutK and/or EutM in L. monocytogenes since both proteins are also hexameric BMC shell proteins with a Pfam00936 domain (see Fig. S1 in the supplemental material).

EA is a valuable carbon source for L. monocytogenes to outcompete other bacteria unable to utilize EA in food environments (27, 28) or the human GI tract, where EA is abundant (5, 6). Our results in defined medium reveal that L. monocytogenes can utilize EA as a sole carbon source via a BMC-dependent *eut* pathway (Fig. 2). Encasing the pathway inside BMCs is essential since this prevents the toxic acetaldehyde intermediate from damaging proteins and RNA/DNA in the cytoplasm (19, 41). The generated reductants are oxidized inside the BMC, while it has been hypothesized that electrons may also be shuttled to the cytosol via specific shell proteins acting as redox carriers (6, 19, 20, 40). Our metabolite analysis showed enhanced flux via the ATP-generating acetate branch in L. monocytogenes and reduced flux via the NAD^+^-regenerating ethanol branch in a 2:1 molar ratio resulting in surplus NADH. In support of the above-mentioned putative electron shuffling from BMCs to the cytosol, using wild-type, EutB mutant, and Ndh2 mutant strains, we identified a link between L. monocytogenes *eut* BMC activity and the recently discovered flavin-based EET system (Fig. 5E) (18). The suggested electron acceptors fumarate and iron are conceivably present in the human intestine and in host cells and have been reported to contribute to L. monocytogenes virulence (17, 18, 42, 43). The identified L. monocytogenes *eut* lmo1185 shell protein belongs to the class of trimeric shell proteins (BMC-T) with two fused Pfam00936 domains. We hypothesize that this predicted PduT-like shell protein with a [4Fe-4S] cluster (39) acts as a redox carrier in this process. Further studies are required to elucidate the role of this *eut* BMC shell protein as a redox carrier.

Taken together, our results provide evidence of anaerobic EA catabolism in L. monocytogenes driven by BMC formation and function, with a crucial role for the flavin-based EET system in redox balancing. These findings provide a new model (Fig. 5E) involving two interconnected cellular subsystems that explains how L. monocytogenes is able to grow and adapt in EA-rich environments like the human GI tract.

## MATERIALS AND METHODS

### Strains, culture conditions, and growth measurements.

All L. monocytogenes strains used in this study are shown in Table S6 in the supplemental material. L. monocytogenes strains were anaerobically grown at 30°C in Luria broth (LB) medium and the defined medium MWB (31). LB and MWB were supplemented with 15 mM EA and/or 20 nM vitamin B_12_ (25). Anaerobic conditions were achieved by using an Anoxomat anaerobic culture system with an environment of 10% CO_2_, 5% H_2_, and 85% N_2_. LB and MWB with 15 mM EA and 20 nM vitamin B_12_ were defined as *eut*-induced conditions, while LB and MWB with 15 mM EA were defined as noninduced conditions. OD_600_ measurements in LB were performed every 2 h during the first 12 h of incubation and at 24 and 36 h. Plate counting in MWB to quantity CFU was performed every 12 h from 0 h to 36 h.

10.1128/mSystems.01349-20.8 TABLE S6 Strains used in this study. Listed are the names of the strains and their sources. Download Table S6, DOCX file, 0.01 MB. Copyright © 2021 Zeng et al. 2021 Zeng et al. https://creativecommons.org/licenses/by/4.0/ This content is distributed under the terms of the Creative Commons Attribution 4.0 International license.

### Construction of strain L. monocytogenes 10403S Δ*eutB*.

The L. monocytogenes Δ*eutB* strain was derived from wild-type strain 10403S (DP-L6253). Gene deletions were generated by allelic exchange using the plasmid pKSV7 (44) with a chloramphenicol resistance gene. Primers used for Δ*eutB* fragment construction and validation of the deletion are given in Table S7. Fragment A and fragment B flanking the *eutB* gene were ligated into the plasmid using Gibson assembly and cloned in E. coli Top10. The plasmid construct was verified by Sanger sequencing. The plasmid was then transformed into E. coli SM10. Transconjugation was performed to integrate the plasmid using a restrictive temperature of 42°C, and colonies that were resistant to streptomycin (200 μg/ml) and chloramphenicol (7.5 μg/ml) were selected. This was followed by passaging in brain heart infusion broth at 37°C with shaking, diluting 1:1,000 every 8 h. The mutant was obtained by screening for chloramphenicol-sensitive colonies followed by colony PCR using validation primers.

10.1128/mSystems.01349-20.9 TABLE S7 Primers used for the construction of strain L. monocytogenes 10403S Δ*eutB*. Listed are the names of the primers and their sequences. Download Table S7, DOCX file, 0.01 MB. Copyright © 2021 Zeng et al. 2021 Zeng et al. https://creativecommons.org/licenses/by/4.0/ This content is distributed under the terms of the Creative Commons Attribution 4.0 International license.

### Analysis of metabolites for EA catabolism.

After centrifugation, the supernatants of the cultures were collected and filtered with a 0.45-μm syringe filter for the measurements. EA (monoethanolamine) at a 1:1 dilution in ethanol was measured by gas chromatography with flame ionization detection (GC-FID), while ethanol and acetate were directly measured by high-pressure liquid chromatography (HPLC). The experiment was performed twice, with three technical replicates per experiment. Additionally, the standard curves of EA, ethanol, and acetate were measured in the concentration range of 0.1, 1, 5, 10, and 15 mM. HPLC was performed as described previously (24). GC-FID conditions for the final method were as follows: a 0.3-μl injection volume, a 260°C injector temperature, an 1177 injector, an SGE column (BP5, Melbourne, Victoria, Australia) focus liner, a 1:10 split, a 335°C detector temperature, and an electronic flow controller delivering 2.8 ml/min helium carrier gas with a 2.0-lb/in^2^ pressure pulse for 0.25 min after injection. The retention time of EA was 11 min, and the total run time was 50 min (45).

### Proteomics.

L. monocytogenes EGDe cultures were anaerobically grown at 30°C under *eut*-induced and in noninduced conditions. Samples were collected at 12 h for LB and at 24 h for MWB to obtain early-stationary-phase cells. The samples were then washed twice with 100 mM Tris (pH 8). About 10 mg (wet weight) of cells in 100 μl 100 mM Tris was sonicated for 30 s twice to lyse the cells. Samples were prepared according to the filter-assisted sample preparation (FASP) protocol (46). Each prepared peptide sample was analyzed by injecting 18 μl into a nanoscale liquid chromatography-tandem mass spectrometry (nanoLC-MS/MS) system (Thermo nLC1000 instrument connected to an LTQ-Orbitrap XL instrument) (24). LC-MS data with all MS/MS spectra were analyzed with the MaxQuant quantitative proteomics software package as described previously (47). A protein database with the protein sequences of L. monocytogenes EGDe (UniProt identifier UP000000817) was downloaded from UniProt. Filtering and further bioinformatics and statistical analyses of the MaxQuant ProteinGroups file were performed with Perseus (48). Reverse hits and contaminants were filtered out. Protein groups were filtered to contain minimally two peptides for protein identification, of which at least one is unique and at least one is unmodified. Also, each group (*eut*-induced and noninduced control groups) required three valid values in at least one of the two experimental groups. The volcano plot was prepared based on the Student’s *t* test difference of *eut*-induced/noninduced groups.

### Transmission electron microscopy.

L. monocytogenes EGDe cultures were grown anaerobically at 30°C under *eut*-induced or noninduced conditions. Samples were collected at 12 h of incubation for LB (early stationary phase). About 10 μg (dry weight) of cells was fixed for 2 h in 2.5% (vol/vol) glutaraldehyde in 0.1 M sodium cacodylate buffer (pH 7.2). After rinsing in the same buffer, postfixation was done in 1% (wt/vol) OsO_4_ for 1 h at room temperature. The samples were dehydrated by ethanol and then embedded in resin (Spurr HM20) for 8 h at 70°C. Thin sections (<100 nm) of polymerized resin samples were obtained with microtomes. After staining with 2% (wt/vol) aqueous uranyl acetate, the samples were analyzed with a Jeol 1400 plus TEM with the 120-kV setting.

### Ferrozine assay of ferric iron reductase activity.

L. monocytogenes cells grown overnight in LB medium were washed with phosphate-buffered saline (PBS) twice, normalized to an OD_600_ of 0.2, and resuspended in fresh MWB medium supplemented with 50 mM ferric ammonium citrate for anaerobic inoculation. L. monocytogenes wild-type and mutant strains grew anaerobically for 24 h in MWB with 15 mM EA and 20 nM vitamin B_12_ (*eut* induced), 15 mM EA (noninduced), 15 mM glucose (positive control), or no added substrate (blank control). Assays were initiated by adding 100 μl of MWB cultures from a 24-h anaerobic inoculation to 100 μl demineralized (demi) water with 4 mM ferrozine, and cultures were then spectrophotometrically measured by the OD_562_ as described previously (18). OD_562_ measurements were made immediately after the initial mixture of MWB cultures and ferrozine.

### Bioinformatics analysis. (i) Secondary structure of N-terminal peptides.

The N-terminal secondary structures of all *eut* genes were determined by a neural network secondary structure prediction server called Jpred4 (49), as described previously (24). The input to the Jpred4 online server was the 50 N-terminal amino acids of each protein. Jnetconf is the confidence estimation for the prediction, with high scores indicating high confidence. Jnetsol25 is solvent accessibility, where B means buried and “−” means nonburied at a 25% cutoff.

### (ii) Venn analysis and STRING networks analysis of proteins.

The protein identifiers of significantly changed proteins from Tables S4 and S5 were uploaded to the BioVenn online server (50) with the default setting to generate Venn diagrams. Overlapping proteins from the Venn diagram were transferred to the STRING online server (51) for multiple-protein analysis of functional interactions using sources such as coexpression, genomic neighborhood, and gene fusion.
