# Supplementary figures and images for: Bacterial Microcompartments Coupled with Extracellular Electron Transfer Drive the Anaerobic Utilization of Ethanolamine in Listeria monocytogenes
Source: mSystems. 2021 Apr 13;6(2):e01349-20. doi: 10.1128/mSystems.01349-20 (PMC8547011; doi:10.1128/mSystems.01349-20)

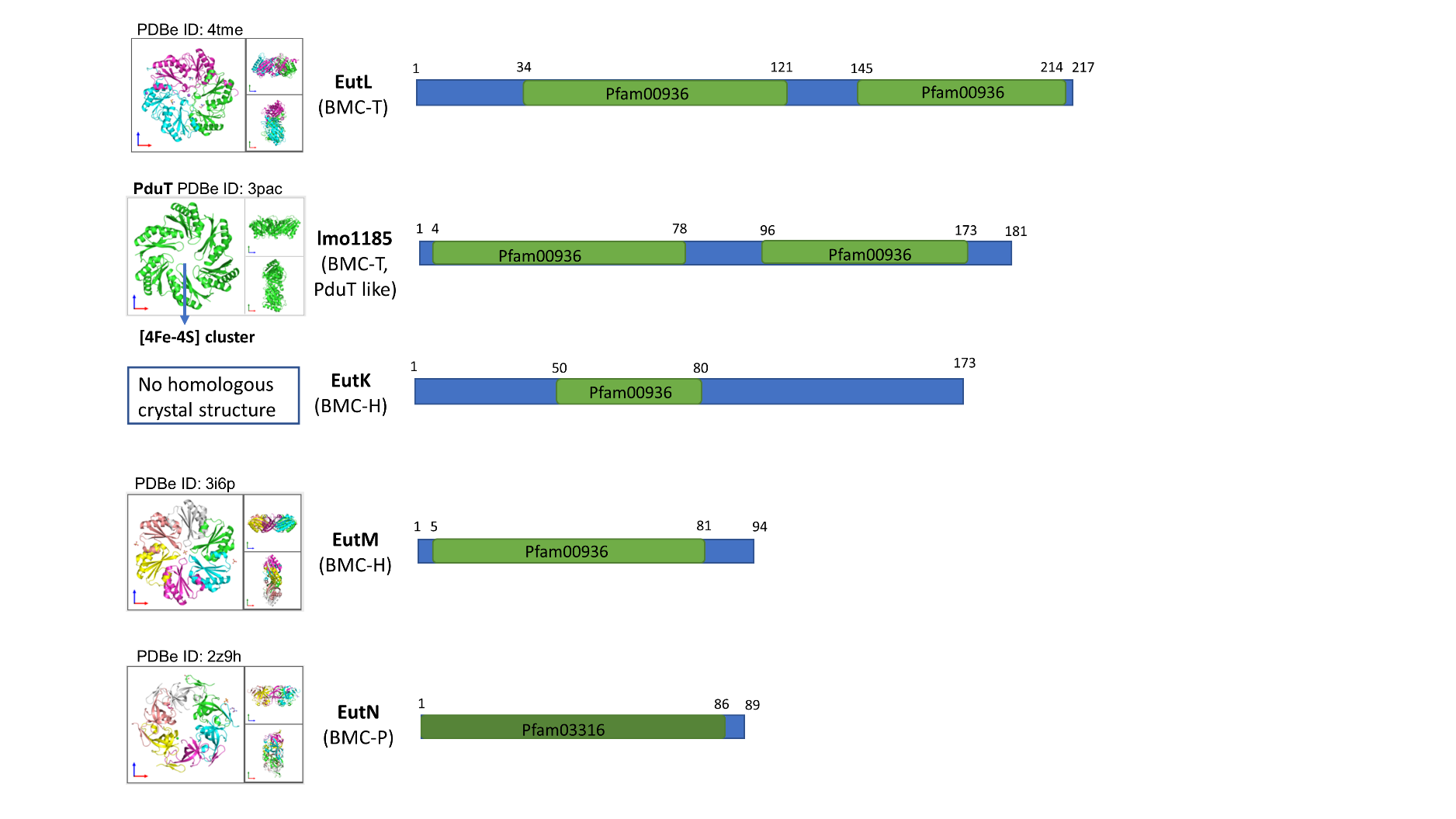

Supplement: FIG S1 [file msystems.01349-20_sf001.docx]

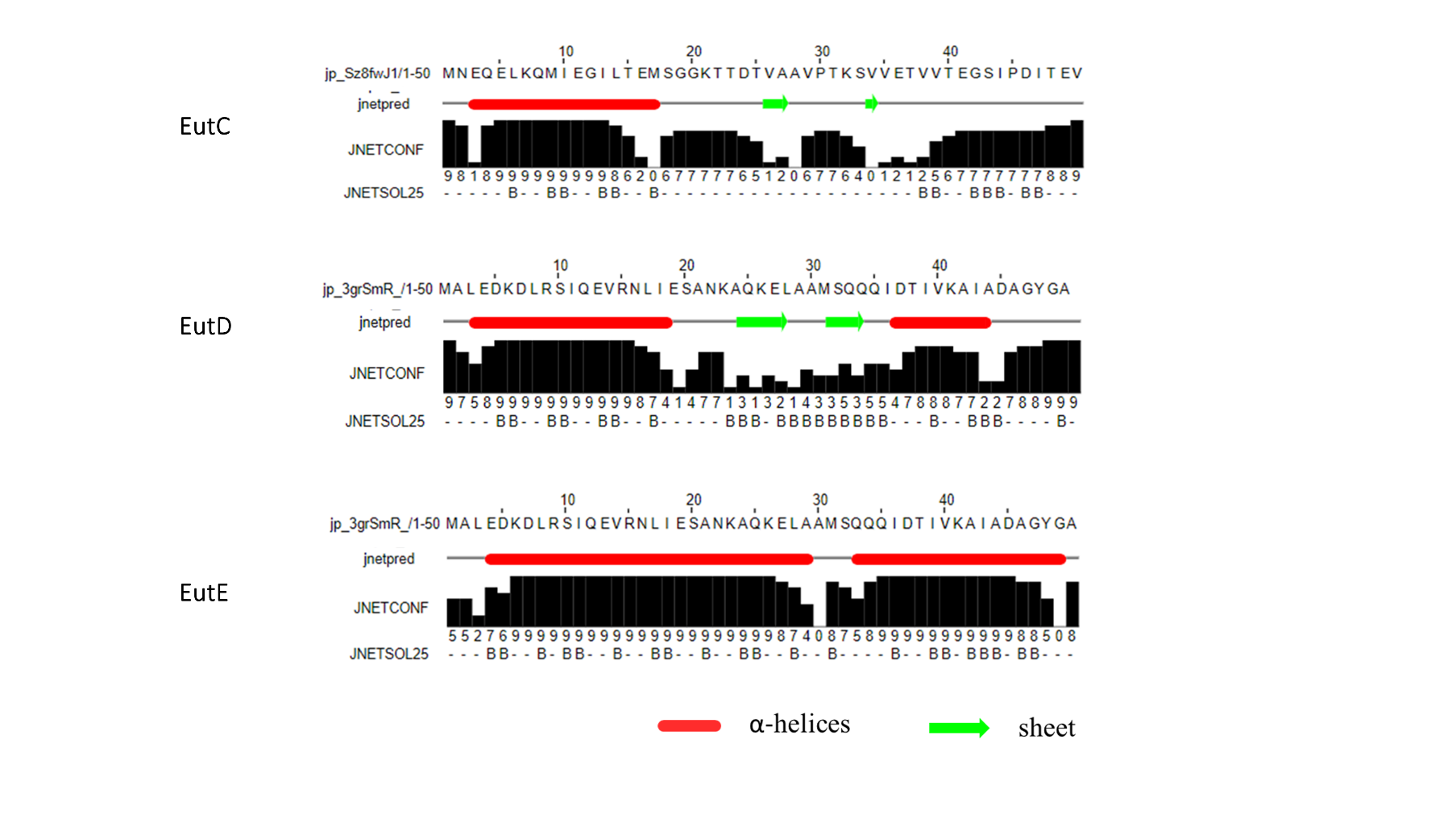

Supplement: FIG S2 [file msystems.01349-20_sf002.docx]
