# Supplementary material for: Bacterial Microcompartments Coupled with Extracellular Electron Transfer Drive the Anaerobic Utilization of Ethanolamine in Listeria monocytogenes
Source: mSystems. 2021 Apr 13;6(2):e01349-20. doi: 10.1128/mSystems.01349-20 (PMC8547011; doi:10.1128/mSystems.01349-20)
Supplement: TABLE S6 [file msystems.01349-20_st006.docx]

Supplementary Table 6.

| Strain | Source |
| --- | --- |
| *Listeria monocytogenes* EGDe | ATCC® BAA-679™ |
| *Listeria monocytogenes* 10403S | Light SH, et al. 2018. |
| *Listeria monocytogenes* 10403S ∆eutB | This study |
| *Listeria monocytogenes* 10403S ∆ndh2 | Light SH, et al. 2018. |
