# Supplementary material for: Bacterial Microcompartments Coupled with Extracellular Electron Transfer Drive the Anaerobic Utilization of Ethanolamine in Listeria monocytogenes
Source: mSystems. 2021 Apr 13;6(2):e01349-20. doi: 10.1128/mSystems.01349-20 (PMC8547011; doi:10.1128/mSystems.01349-20)
Supplement: TABLE S7 [file msystems.01349-20_st007.docx]

Supplementary Table 7.

| Primer | Sequence (5' to 3') |
| --- | --- |
| EutB Fragment A (forward) | ATGGGGTCCAGCGGCGCTGGATCCTTGCAGACTGTATTTCAACTGGTG |
| EutB Fragment A (reverse) | AGGATTTATCGAATGATTTTAAAAACGAATTCTATTTTCCTAAAATAA |
| EutB Fragment B (forward) | ATGATTTTAAAAACGAATTCTATTTTCCTAAAATAAGGAAGGGAGG |
| EutB Fragment B (reverse) | TACAAATCGTTGTTGGTGATGGACGCTGCAGGAGGCAGTGGAGCGAGC |
| ΔEutB validation (forward) | GCTTGCTTAGATATTGGTGGTCG |
| ΔEutB validation (reverse) | GAGGAACTAAGCGATGAACGAACA |
